# Capturing Memories for Children With Cancer in a Low-Resource Setting

**DOI:** 10.1200/GO.23.00001

**Published:** 2023-02-28

**Authors:** Allison Silverstein

**Affiliations:** ^1^ Department of Pediatrics, Baylor College of Medicine, Houston, TX; ^2^ Section of Palliative Medicine, Department of Pediatrics, University of Colorado School of Medicine, Aurora, CO

I was the paparazza, capturing salient moments from our program's “Palliative Care Day” where children with cancer and their guardians played games, completed artwork, sang and danced, and enjoyed meals together. It was a precious day for these children with life-limiting disease to shed the weight of their diagnoses and instead share laughter and joy with one another. As a pediatric resident on a global health year in Malawi, I was invited to document our team's activities with the intent to share with potential donors. However, with a click of the camera's button, I realized the opportunity for an unintended greater impact. I scrolled through the day's pictures and could not help but think the recipients of the pictures should not be strangers, but instead the families or even children themselves. Although families had already provided consent for each picture, they never expected to see them.

Pictures capture moments in ways words cannot describe. Coming from a Western society where we celebrate and honor life in pictures, I wondered what happens when you do not have a camera or phone capable of capturing these events. What visual memories do you have when your child dies? Does it feel differently when remembering a lost child without pictures to look at? Do vivid memories fade and, in time, make it difficult to imagine your child's face?

As I reflected on this, I acknowledged the overwhelming frequency of childhood cancer death in our setting—in contrast to a >80% survival rate for childhood cancer in the United States,^1,2^ the childhood cancer mortality rate is estimated to be as high as 90% in sub-Saharan Africa.^3^ Most of these children present with advanced disease, where disease-directed treatment is less likely to be effective,^4^ and limited availability of medical and supportive care further contribute to poor outcomes.

Although progressive medical infrastructure has sprouted across regions of sub-Saharan Africa to help address these disparities, widespread gaps exist in interdisciplinary services. Families of children with cancer face substantial psychosocial, emotional, and spiritual distress. Many families are fortunate to have robust community support, but we must consider how we, as a medical system, can further support families. Our role includes providing comfort to families, especially when curative medical therapy is not an option and a child's final days near. We must integrate humanities and holistic support for our families as we scale up global health programs, just as is already done in high-income settings.

So, when I set my camera aside, I earnestly turned to my local colleagues for their counsel. They grinned as they confirmed the potential value of my blossoming idea. I went to a nearby store where I printed the pictures and purchased basic supplies—glue, string, tape. We collected old boxes from prior hospital pharmacy deliveries and bought local vibrantly colored fabric—chitenje—from the market. From these materials, our first frame was designed. These local materials were obtained on a minimal budget.

**FIG 1:**
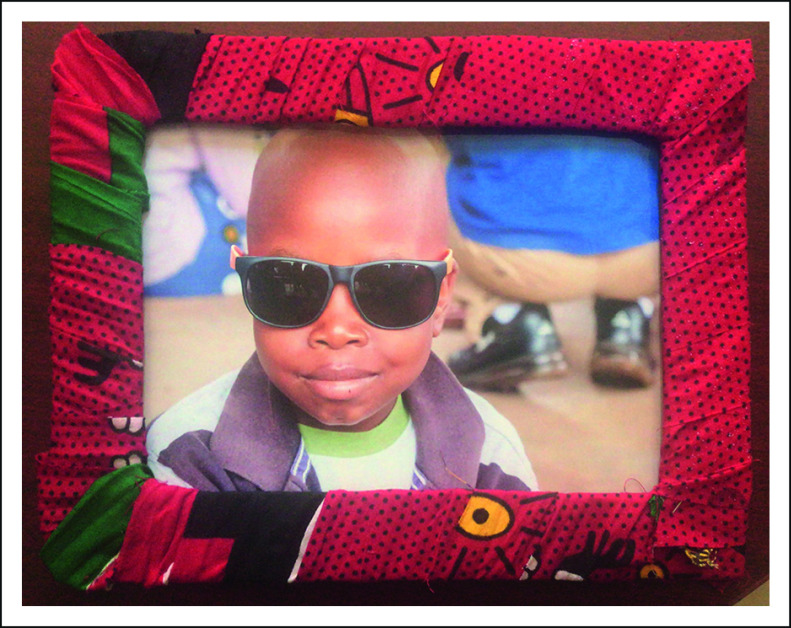
Original frame and picture created as part of the picture program. Consent to share generously provided by guardian.

I shared the first picture and its frame with our social worker who presented the aunt of P with the picture (Fig 1); P had leukemia and had died recently from complications associated with central nervous system disease. In his picture, there he was, coloring during the event we held a few weeks prior. He wore sunglasses and shared that smirk we had all quickly fallen in love with. As she graciously accepted the frame, the corners of P's aunt's mouth turned upwards into a rarely seen smile; she bowed her head silently as we spent a moment remembering P and sharing in his memory.

The next week, I had the privilege of joining our team on a bereavement visit to the home of B's father. B had recently died at home and our team visited to provide grief support and share prayers together. We sat in a circle on well-worn couches and chairs as B's father offered he did not have any physical belongings or keepsakes of his son beyond leftover medical supplies from home wound care management; any clothes or toys were passed along to other children and other families. As he shared with us, he removed a cloth covering their makeshift table to reveal a cardboard box, inside of which he retrieved these remaining medical supplies so they could be given to another family. We pulled out a framed picture of B that was taken before the program had formally started but was printed and framed just as the others. I watched as B's father's eyes welled up with tears in surprise and gratitude; he accepted the gift and stood to shake each of our hands.

One by one, we started taking more pictures. My colleagues explained the idea of the project as we obtained consent from each new family. Often we were met with a bit of initial skepticism but also willingness to participate. Pictures were taken away from the crowded medical wards and instead in courtyards with benches, grass, and trees as possible. As we delivered the first batches of framed pictures to families, the skepticism was quickly replaced with enthusiasm, and families embraced the program. We could not seem to print consent forms fast enough, as caregivers changed outfits, brushed their hair, and sought us on the wards to request portraits. They claimed their pictures like prizes. Some of the children lived to see them. Others died.

The picture project served as emotional support for families, most of whom had or would lose their children. In time, the program transitioned from volunteers constructing frames to caregivers themselves making the frames together; they sat in open green spaces and connected, providing an organic social support system for one another.

With the start of the COVID-19 pandemic, I returned to the United States to continue my training, and my colleagues in Malawi faced new challenges of their own. Just as staffing shifted at my home institution, so too were modifications made in Malawi to optimize patient and team safety. Although our framed photograph program paused similarly to many supportive care programs across the world, months later, my colleague shared a picture with me: a group of caregivers gathered on a lawn, a pile of frames and photographs scattered on the ground, the program restarted, and the memories being created and shared once more.
